# Towards co‐designing active ageing strategies: A qualitative study to develop a meaningful physical activity typology for later life

**DOI:** 10.1111/hex.12686

**Published:** 2018-04-06

**Authors:** Cornelia Guell, Jenna Panter, Simon Griffin, David Ogilvie

**Affiliations:** ^1^ MRC Epidemiology Unit and UKCRC Centre for Diet and Activity Research (CEDAR) University of Cambridge Cambridge UK; ^2^ European Centre for Environment and Human Health University of Exeter Truro UK; ^3^ The Primary Care Unit Institute of Public Health University of Cambridge Cambridge UK

**Keywords:** active living, ageing, intervention strategies, participatory research, UK

## Abstract

**Background:**

Physical activity levels decline in later life despite the known benefits for physical, cognitive and mental health. Older people find it difficult to meet activity targets; therefore, more realistic and meaningful strategies are needed. We aimed to develop a typology of older people's motivations and lifelong habits of being active as a starting point to co‐designing active ageing strategies in a workshop.

**Methods:**

We conducted semi‐structured interviews with 27 participants aged 65‐80 in Norfolk, UK, and participant observation with 17 of them. At a workshop with 13 study participants and 6 government and civil society representatives, we invited reflections on preliminary findings.

**Results:**

Three types were developed. “Exercisers” had engaged in sport and exercise throughout their life but experienced physical ill health and limitations as barriers. “Out‐and‐about‐ers” pursued social engagement and a variety of interests but experienced biographical disruption through retirement and loss of companions that limited social activities in later life. A final type characterized people who preferred “sedentary/solitary” activities. A workshop elicited suggestions for new strategies relating to these types that addressed people's specific motivations. An example was to combine social engagement and physical activity in “dog‐parent”‐walking schemes to link people through shared responsibility for a dog.

**Conclusions:**

We suggest that these potential strategies map more closely onto the everyday life‐worlds in which public health might seek to intervene than common physical activity interventions. Most notably, this means a more differentiated understanding of barriers, and acknowledging that intellectual, social or solitary pursuits can include incidental physical activity.

## 1 INTRODUCTION

Our populations are ageing, and leading active lives is considered a “best buy” for preventing chronic conditions such as diabetes, heart disease and cancers, cognitive decline and dementia as well as social isolation and mental ill health.^1^, ^2^, ^3^, ^4^, ^5^ Many older people, however, find it difficult to meet current physical activity guidelines.^6^ Systematic reviews suggest that, to date, interventions promoting physical activity have typically produced only small or short‐lived behaviour change.^7^, ^8^ Yet these reviews tell us little as to why success has been so limited. Public health intervention strategies tend to target either “at‐risk” individuals, often focused on structured activities (eg, gym referrals), or “the population” at large, for example through health promotion campaigns (eg, to encourage walking) or through providing more supportive physical environments (eg, cycle lanes).^9^ An underlying assumption of most physical activity interventions seems to be that people who are “insufficiently active” might need to learn about the benefits of being active or might require more appealing or accessible opportunities for sports and exercise.

What seems to be missing from the development of many intervention strategies is a greater understanding of people's dispositions, aspirations and life‐worlds that underlie their physical activity patterns. People who are considered not to meet recommended activity levels cannot be assumed to share similar preferences or constraints, and this is where current interventions might fall short. While quantitative physical activity studies generally classify the population into those meeting or failing to meet particular physical activity targets, and some studies attempt to characterize participants into activity profiles (eg, by ability, physical activity domain or demographic characteristics),^10^, ^11^, ^12^, ^13^ these are usually based on people's current (reported or measured) activity level. People subsumed as “inactive”, however, might be so for a variety of reasons, which could not be addressed through the same strategy. Older people might experience a variety of barriers from physical limitations to social isolation and neither necessarily related to their attitudes towards physical activity. Developing a more in‐depth insight into people's experiences would entail moving beyond a broad‐brush, generic approach to individuals, communities or populations to actively involve people in research that aims to understand how health behaviour is shaped by their biographies and deeply embedded in their social and physical environments.^14^ This might also help to interrogate the inherent assumptions underlying common intervention strategies and could inform future strategies that map more closely onto the everyday life‐worlds in which 1 might seek to intervene.

The overall aim of this study was to better understand the varied experiences of declining physical activity in later and to develop a differentiated approach to addressing barriers in more realistic and meaningful ways. First, we undertook qualitative research to explore lifelong practices and aspirations of active ageing, developed a motivation‐based typology for being active and described respective barriers to staying active into older life. In a second part to this study, we held a workshop in which we invited participants to contribute to the interpretation of their data. The workshop was conceived of as an exercise in collaborative analysis and reflection to jointly discuss new directions for intervention strategies to promote active living.

## 2 METHODS

### 2.1 Participants

This qualitative study was embedded in a larger epidemiological project on physical activity in later life and its health outcomes, which in turn made use of data from the European Prospective Investigation into Cancer and Nutrition (EPIC)‐Norfolk study.^15^ We purposefully sampled a diverse, information‐rich sample of 27 women and men, aged 65‐80 years, classified as belonging to a professional or manual occupational class, living alone or cohabiting, in urban or rural neighbourhoods in Norfolk, UK, and by physical activity level. The EPIC‐Norfolk study coordination team initially mailed 32 potential participants, and 22 agreed to take part; another 8 were invited in a second round of recruitment to fill under‐represented categories (inactive, manual occupational class and oldest age group), of whom 5 responded positively.^16^ For purposive sampling, we had access to EPIC participants' demographic data and their latest accelerometer measures of physical activity (see Table 1) which we used to sample participants belonging to a more (but not the most) active group or a more (but not the most) inactive group. This should enable us to gain insights into the experiences of both older people who experienced barriers or had the motivation to staying active and those who did not; we assumed that the most active and inactive might have very particular experiences such as severe ill health or unusual sporty pastimes. As these objective measures would form the basis of many of the epidemiological analyses of the overall project, we aimed to see in what way these might map onto people's everyday experiences.

**Table 1:** Participants by type

Activeness type	ID	Activity level^a^,^b^	Gender^a^	Age^a^	Occupation^c^	Location^a^	Living status^a^	Comment^d^
Exerciser	09	Active	Man	70‐74	Manual	Rural	Living alone	Also out‐and‐about‐er; still in paid work
	11	Active	Man	70‐74	Professional	Urban	Cohabiting	Also out‐and‐about‐er
	12	Active	Woman	70‐74	Professional	Urban	Cohabiting	Also out‐and‐about‐er; volunteers, walks for transport; reported chronic illness
	16	Active	Woman	70‐74	Manual	Rural	Cohabiting	Also out‐and‐about‐er; has dog; reported musculoskeletal limitations
	22	Active	Woman	65‐69	Professional	Rural	Cohabiting	Also out‐and‐about‐er; works; reported partner's ill health
	15	Active	Woman	75‐80	Manual	Rural	Living alone	Not active at time of interview; lost dog; reported musculoskeletal limitations
Out‐and‐about‐er	03	Active	Man	65‐69	Professional	Rural	Cohabiting	Reported partner's ill health
	06	Active	Woman	65‐69	Manual	Urban	Living alone	Reported partner's ill health
	07	Active	Man	65‐69	Manual	Urban	Cohabiting	Walks for transport
	10	Active	Man	65‐69	Professional	Rural	Living alone	Still in paid work; reported chronic illness
	14	Active	Woman	75‐80	Professional	Rural	Living alone	Still in paid work, has dog
	01	Inactive	Woman	65‐69	Professional	Rural	Cohabiting	Still in paid work; reported musculoskeletal limitations
	02	Inactive	Man	75‐80	Professional	Rural	Living alone	Volunteers
	05	Inactive	Woman	65‐69	Professional	Urban	Living alone	Still in paid work
	17	Inactive	Man	70‐74	Professional	Rural	Cohabiting	Reported musculoskeletal limitations
	20	Inactive	Man	75‐80	Manual	Rural	Living alone	
	24	Inactive	Woman	75‐80	Manual	Rural	Living alone	Reported musculoskeletal limitations
	25	Inactive	Woman	75‐80	Manual	Rural	Cohabiting	Reported chronic illness
	27	Inactive	Man	75‐80	Manual	Rural	Cohabiting	Volunteers, walks for transport; reported chronic illness
Sedentary	04	Active	Man	65‐69	Professional	Urban	Living alone	Still in paid work, walks for transport; reported partner's ill health
	08	Active	Man	70‐74	Manual	Urban	Living alone	Volunteers, walks for transport; reported musculoskeletal limitations
	19	Active	Woman	65‐69	Professional	Urban	Living alone	Volunteers, walks for transport
	13	Inactive	Man	75‐80	Professional	Urban	Living alone	Volunteers; reported musculoskeletal limitations
	18	Inactive	Man	70‐74	Professional	Urban	Cohabiting	Volunteers; reported partner's ill health
	21	Inactive	Woman	65‐69	Manual	Rural	Cohabiting	Cares; reported partner's ill health
	23	Inactive	Man	75‐80	Manual	Urban	Cohabiting	Reported chronic illness
	26	Inactive	Man	75‐80	Manual	Urban	Living alone	Reported chronic illness

a From European Prospective Investigation into Cancer (EPIC)‐Norfolk fourth health check data (2013‐2015).

b From Actigraph accelerometer counts, decile 7, 8 and 9 for more active group, decile 2, 3 and 4 for more inactive.

c From EPIC‐Norfolk baseline data (1993‐1997).

d From qualitative study.

A year on, we contacted all 27 participants, of whom 13 were able to attend a participant workshop. We also recruited 6 expert stakeholders through direct invitation from our existing networks and snowballing through personal recommendation, all of whom agreed both to attend the workshop and to be interviewed afterwards. These stakeholders were carefully chosen to represent a variety of local and national, practice and policy interests and comprised 2 county council public health officers, 3 regional civil society representatives and 1 national government representative either holding remits in physical activity and/or older people. We aimed for a small enough group of expert stakeholders not to intimate our lay participants.

### 2.2 Data collection

*Semi‐structured interviews*: Between September 2014 and March 2015, 27 semi‐structured interviews, lasting 20‐60 minutes, were conducted in participants' homes by 1 of 2 experienced qualitative researchers. The interview guide covered questions about their everyday activities and motivations; their lifestyle opportunities, choices and motivations across the seasons and the life course; and their aspirations into older age. All interviews were audio‐recorded and accompanying field notes taken.
*Participant observations*: We also conducted “semi‐structured” participant observations to seek reflections on embodied experiences less easily articulated in interviews. The participant observation sessions with 19 of the participants were organized at the end of each interview and undertaken with the same researcher who conducted the interview. We called these “semi‐structured” participant observations, as the researchers joined participants for a habitual activity. The participant observation sessions, which included yoga, walks, shopping trips, gardening, art classes and bus rides as well as visits to workplaces, lasted 1‐3 hours and were written up in ethnographic field notes.
*Participant workshops*: In April 2016, we held a 2.5‐hour lunchtime workshop at a central location. After a presentation of our summary findings, workshop attendees were invited to share reflections of both analytical concepts and practical intervention ideas as users, facilitators or commissioners of such initiatives. Small group discussions concentrated on immediate feedback on the study results and the proposed typology and then moved the debate forward towards feedback on common strategies such as media campaigns, exercise classes and walking groups, how these resonated with their own experiences and whether they would appeal to them or peers in their age group. Finally, we explored initiatives the participants would like to see developed and how they might suggest the policymakers and practitioners present could promote active living.
*Follow‐up interviews*: The expert stakeholders were then invited for follow‐up telephone interviews (lasting between 25 and 45 minutes) to elicit further reflections on the study results and analytical thinking, but also to gain feedback on the workshop outcomes, in what way these resonated with their own work, and in what way they might use insights from the workshop in their practice.

### 2.3 Data analysis

The initial verbatim interview transcripts were coded for thematic analysis, which involved familiarization with the textual data through repeated reading, identifying codes and synthesizing larger thematic categories. The coding was guided pragmatically by the research objectives but also allowed for inductive analysis of unanticipated topics or meanings. The ethnographic field notes served to triangulate the findings; they were not openly coded like the transcripts, but used to help at the later stage of analysis when codes were synthesized into categories, further exploring how categories played out differently in different situations or participants. We then further analysed our main findings (summarized in the concept of “activeness”)^16^ to develop the typology and reflected on these emerging insights during the participant workshop and follow‐up expert interviews. The ethnographic field notes from the workshop and interview transcripts were again thematically analysed and used to refine analytical categories. Data management and analysis were aided by the qualitative data analysis software NVivo 10.^17^

The study's 2 parts of qualitative data collection and the follow‐up workshop and its embeddedness in a larger EPIC study enabled us to triangulate our findings in various ways. We were able to compare in what way the emergent qualitative typology matched the participants' objectively measured physical activity on which recruitment was based in particular the (see Table 1). We were also able to compare the initial interview responses with participants' comments on their synthesis and interpretation during the workshop.

The study received ethical approval from the NRES Committee South Central – Oxford C (14/SC/1047), and all participants gave their written informed consent. An amendment with separate written consent was sought for the participant workshop and follow‐up interviews with expert stakeholders. Study results shared during the workshop were carefully anonymized.

## 3 RESULTS AND DISCUSSION

### 3.1 Developing types of activeness to understand differential barriers

We developed 3 motivational types of activeness, which included the disposition to be “exercisers,” “out‐and‐about‐ers” and the “sedentary/solitary” (see Table 1). We understand these types in the tradition of Weber's approach of ideal types, deriving them from, but not representing, social reality; instead, ideal types enable interpretation as analytical constructs with distinct explanatory characteristics and enable comparison within and across types.^18^ This is important to note, as it means that participants could fall into several or all of the activeness types.

*The “exerciser”*: Some of our participants described themselves as sporty and enjoying exercise, often in reference to their biographies. Developed through early life, adapted through life events such as having children or entering and leaving the workforce, engagements with sport and exercise varied but were maintained habitually throughout life into older age. For example, a participant recounted that he was introduced to ball games at school, enjoyed being part of a team at the workplace and later sought a club in his neighbourhood to continue his pastime into retirement.

I played football, cricket, outdoors, badminton …, tennis … [11, man, 70‐74, active]

All my life I've played tennis, since I was a little girl … always sporty, yes, yes. [15, woman, 75‐80, active]

During the workshop, some participants talked less about their enjoyment of exercise, sport or competitiveness and more about their explicit motivation to engage in health‐enhancing activities, for example to improve posture, strength or endurance and therefore saw themselves as belonging to this type.

Some “exerciser” participants experienced barriers such as declining physical health and mobility, which prevented them from being as active as they would have liked. They attempted to stay as active as possible and to continue lifelong sports and pastimes, for example by changing towards lower impact activities (from sports to walking) or lower intensity activities (from tennis to short tennis to game console tennis; from cricket to bowls).

… I play short tennis and I play bowls a lot and I walk. I played golf until two years ago and tennis and badminton. [Q: Very active, so have you always been active?] Yes, … [I'm] very sporting. Joints don't allow you after a while, knee joints, anyway. … But … it's something I've always done and so I love doing it. [12, woman, 70‐74, active]

The quantitative measures for these participants show that all of our “exercisers” fell in the “active” category (objectively defined using accelerometer step counts; see Table 1). We should also note that most of our “exercisers” also fell into the category of “out‐and‐about‐ers”.

*The “out‐and‐about‐er”*: Many participants stayed active in a much broader way than by being explicitly physically active. This was particularly described as the motivation to be engaged in many social or intellectual activities. An “out‐and‐about” participant would, for example, list a variety of activities across a typical week, ranging from housework and gardening and meeting up with friends to looking after grandchildren or elderly parents, rarely missing out a day of the week in this tight scheduling. Some of these participants had less varied interests but were still leading busy work lives or were walking daily as their main means of transport.

I've always been on the go, I've never, ever been slow. I just am not that type, you know, I don't like sitting … and just watching telly. [6, woman, 65‐69, active]

[I'm] Active, I can't sit down for long, my son gets very angry with me, ‘Dad you're turning old', I said ‘So what? Do you want me to sit down', ‘Yes', I said ‘No, I can't'. [16, man, 75‐80, inactive]

The participants quoted here used to do manual jobs in their working lives, and their perspective resonates with previous research that former manual workers may be more inclined towards purposeful activities in later life in preference to more leisure‐based activities.^19^ This has been described elsewhere as the “busy ethic”, the strong motivation of continuing a work ethic into retirement.^20^ However, in our sample, we could not find any difference between occupational classes in this respect, and those previously working in professional occupations also talked about the importance of staying busy.

The “out‐and‐about‐ers” also experienced physical limitations as a main barrier to continuing lifelong activities. For example, a participant struggled to keep up bird watching in terrain that was now too uneven for them. Another explained that she stopped walking with a walking group because she would have had to keep up with the length of the walk and pace of the group. In the case of the “out‐and‐about‐ers”, though, their narratives were less about losing the ability to be physically active and more about losing out on social engagements and pastimes beyond sport. Loss of previous activities included the loss of activity companions in people's social worlds, through ill health or death of partners, separation from partners, children moving away, grandchildren growing up and the death of dogs. All these were narrated as reasons for current sedentary living or for attempting to find new ways of staying socially involved.

If I retired I'd have to do something that involved people because I do like being with people. So, you know, I'd have to find something that I could do that involved meeting others or, you know, sort of working with others in a voluntary capacity or something but I couldn't stay at home and be insular, you know, it's just not my nature. [14, woman, 75‐80, active]

More broadly, limitations included experiencing retirement as a very busy life stage that did not leave much time for fitting in activities; or simply that for some of our participants, retirement meant that previous activities had stopped. However, about a third of our “out‐and‐about‐ers” were also objectively classified into the more active group (see Table 1), thus showing that being socially active could be a viable strategy for active ageing.

*The sedentary/solitary*: Finally, some participants—or friends or family they were characterizing—were neither particularly fond of sports and exercise, nor enjoyed social activities, nor had a busy schedule of commitments. They appreciated more quiet moments of rest, or at home, sometimes related to having retired from busy or manual working lives. These participants talked about avoiding the stress of too many engagements or of trying to avoid replicating their busy work lives in retirement. Some, in their disposition, were particularly put off by group activities, preferring solitary pursuits over those requiring social interaction, and others struggled to find the motivation to be physically or socially active.

I feel sorry for those people who have worked so hard and don't know how to use retirement and leisure time, but the nice thing about being retired is that you can pick and choose what you want to do. [13, man, 75‐80, inactive]

I think I'm relatively inactive actually. … We … lost our dog, which was always a perfect excuse to have to go out to take the dog for a walk, and my partner used to be very active as far as walking was concerned and now that's gone [since being wheelchair bound]. The motivation isn't there, I don't feel motivated so much to do things physically on my own… [21, woman, 65‐69, inactive]

Interestingly, some of those classified by us as belonging to the sedentary/solitary type remained relatively physically active because their daily activities involved continued social responsibilities such as caring for grandchildren, siblings, parents or neighbours (see Table 1).

### 3.2 Intervening in meaningful ways to promote active living

As described above, our typology reflects people's preferred ways of being active and does not necessarily map onto current activity levels. Common quantitative physical activity types or profiles tend to classify physical activity using (self‐reported or objective) measures of the frequency or intensity of current behaviours; such types include “active,” “inactive” and “sedentary” as well as more differentiated alternatives, for example “active but sedentary” (gym goers in sedentary jobs).^13^ The distinction between present levels, past disposition and future aspirations seems crucial, however, as the barrier for such physical activity is not a lack of motivation but a lack of opportunity or capability.^21^ In fact, the distinction in our typology between physical, social and intellectual motivations and activities can aid reflection on typical (formal) intervention strategies to promote active living. “Classic” behavioural interventions tend to be framed within models that assume reasoned and planned behaviour based on the combination of a positive attitude, perceived social acceptability and capability to engage in a certain behaviour.^22^ This maps somewhat onto our notion of motivation, a lifelong disposition, sense of self and a deliberate aspiration of how to spend the everyday^23^; however, critical appraisals of behaviour change models suggest that attitudes and intention are not on a linear path towards individual behaviours, and barriers to certain behaviours often lie beyond personal attitudes in the social or physical realms.^14^ More sophisticated intervention models acknowledge the importance of addressing the combination of capability, motivation and opportunity as these shape behaviour.^24^

“Exercisers” in this study certainly enjoyed exercise all their life and did not need to be convinced thereof (eg, in social marketing campaigns); yet they experienced other barriers which prevented them from being active and would need to be addressed (eg, affordable or low‐impact exercise opportunities, or age‐specific classes). At first glance, the aim of typical exercise‐based interventions is to make exercise more acceptable for “non‐exercisers”, for example by “disguising” workouts as dance (Zumba) or making exercise groups age‐ or gender‐specific and therefore perhaps less intimidating.^25^ Through the lens of our ideal types of activeness, however, they might not actually attract “non‐exercisers” but simply target “low‐hanging fruit” by providing more variety or adapted versions of activities for exercisers. To put simply, someone who considers herself “not a gym person” but prefers activities in nature will not necessarily feel more positive about a women‐only gym. Targeting the population group of “women”, here, fails to account for underlying varying dispositions.

While many “out‐and‐about‐ers” might not be attracted by the offer of structured exercise, they might be more likely to appreciate safer walking routes or more convenient public transport to support their way of being active. Indeed, population strategies to promote active living now often include infrastructural improvements, for example providing pedestrian crossings, cycle routes or green spaces.^26^ “Out‐and‐about‐ers” with a busy schedule of activities might be supported by including physical activity in even sedentary engagements such as a theatre visit, if this could be reached by a safe and convenient walk. Such environmental interventions are sometimes based on the assumption: “build it and they'll use it”^27^; if only physical or social environments were more supportive (eg, more pedestrian‐friendly), people would realize that it is both feasible and, trying the new activity, even enjoyable. However, similar limitations might apply. Supportive environments for travel may speak best to those who travel. And intervention that promotes physical activity on a population level, for example through cycling networks, might create disabling environments for older people.

### 3.3 Co‐developing new strategies for staying active in later life

During our workshop, we invited participants to join our thinking. Discussions focused more on practical ideas participants would enjoy in their everyday lives and less on the abstract fitness‐for‐purpose of the ideal types and their relation to intervention frameworks. Three of the ideas that emerged were—if not entirely novel—very striking.

First, our finding that people like purposeful activities to keep active in later life strongly resonated with all workshop discussion groups. Ideas included historical or botanical walks, that is walking trails or walking groups that included routes along historical sites or with an emphasis on local flora. While this idea was developed at one of the discussion tables, others responded enthusiastically; and our study had shown that much walking had been done for a variety of purposes, for example to socialize with friends, go bird watching, see formal gardens or walk the dog.

Second, the discussions picked up on the importance of the social world in which activeness takes place. Activity companions in the broadest sense included friends, family and notably dogs and were considered very important motivators. A second idea that emerged during the workshop was that of a dog‐walking partnering programme. Schemes for borrowing dogs for walking already exist in a variety of forms, mainly as online platforms, but our participants were keen to have a programme that would help connect older people who had lost dogs or did not want to get a new dog at their advanced age, with specific younger dog owners who struggled to make enough time for their dog during their working life.

Third, study participants at the workshop much preferred intergenerational to age‐specific programmes. While some had no family nearby (or at all), and others experienced that their grandchildren grew up too quickly to be a viable source of activity for them in the longer term, they would nonetheless appreciate more social interaction across generations. A third idea was therefore to enable older people to learn from younger people how to use mobile phone apps to track their activities.

These ideas spanned across the activeness types. Historical or botanical walks provide straightforward exercise, social interaction and intellectual stimulation. Similarly, a “dog‐parent” programme could match a variety of dispositions; it could be solitary or social (depending on the relationship between the dog sharers), an opportunity for exercise or simply to get out of the house. Finally, the “apps class” would allow for social engagement, intellectual learning and ultimately either enable the tracking of exercise aspirations or support solitary activity.

The expert workshop attendees from policy and practice were particularly struck by having their assumptions challenged through these workshop discussions.

I think one of the clear messages that I got from a kind of commissioning perspective was that we make too many assumptions around what we think that older people around physical activity need but that isn't possibly the actual thing that they want. So for example we were talking about … group exercise classes and my assumption would have always been to build interventions or projects around specific age groups and actually they said no. They were … very much for intergenerational work around mixing age groups and that would actually be motivating for them to join in because they enjoy learning from younger people. [local government stakeholder]

Moreover, the expert stakeholders were struck by the relatively low priority of “health” as an explicit motivator and summarized the workshop suggestions as “health by stealth”, being engaged in physical activity for reasons other than trying to be physically active. “Out‐and‐about‐ers” engaged purely incidentally in physical activity through taking part in their various interests and social relationships. However, the motivation to be active beyond physical activity as its main health benefit was even applicable to the exercisers; they seemed to gain enjoyment from movement, competitiveness or a challenge. These discussions of incidental physical activity then sparked ideas in some of them of “sneaking” active living programmes past funders “disguised” as dementia or social isolation programmes. There is an increasing recognition that public health strategies can focus on “non‐health” outcomes such as sustainable travel and still be able to reap its health cobenefits.^28^ Well‐being is sought for a broad range of motivations, interests and experiences, and strategies such as a community theatre group for older people might be able to address a range of public health issues such social isolation, cognitive decline and physical inactivity.

It's about knowing what the drivers are for those councillors or those politicians and so often socialisation, loneliness are big things, so how do we make sure we use physical activity as a vehicle to overcome those? And maybe it's about us just … mapping each of their priorities back to actually physical activity can help to solve this. [local government stakeholder]

## 4 CONCLUSION

Common quantitative physical activity research either classifies people into achieving particular physical activity levels or into profiles describing ability, socio‐demographic characteristics or physical activity domains such as particular sports, transport and work or domestic activities.^10^, ^11^, ^12^ To better understand how older people can be supported in staying active, we have developed a typology to help distinguish older people's motivations: those who have always enjoyed sport and exercise, those who are active in terms of social engagements and varied interests, and those who prefer more sedentary or solitary pursuits. We suggest that the limited success of many strategies in shifting population physical activity patterns might partly reflect a poor match with people's dispositions and aspirations; these interventions merely address the problem—low physical activity levels—without addressing specific barriers people experience in following their motivations due to challenges such as ill health or loss of activity companions.

To develop this motivational typology, we combined in‐depth interviews with participant observation, for more in‐depth understandings of participants' experiences, and workshop discussions for a combination of informal and formal reflections on our findings. Important for us was the participatory nature of this study, to allow participants to show and share with us their common activities and pastimes, and the social and physical environments in which these take place. The participatory workshop then enabled us to share our preliminary findings and emerging conceptual ideas, gain feedback for their usefulness from stakeholders in policy and practice and encourage dialogue between lay and expert participants.

Our participants identified ideas that can speak to a variety of dispositions and respective barriers, for example by enabling both physical and intellectual activity, solitary or social pursuits, and often promoting physical activity as a secondary “co‐benefit”. Intervention strategies increasingly acknowledge these issues, for example by adding “opportunity” to behaviour change models^24^ and increasingly targeting broader concepts of physical activity to reach out beyond exercisers.^26^ Including participants more effectively in developing policy and practice, and recognizing the importance of people's biographies and social worlds, might be equally important in identifying meaningful active living strategies.

## CONFLICT OF INTERESTS

The authors declare no conflict of interests.

## AUTHORS' CONTRIBUTIONS

CG, SG and DO were involved in the study conception and design. SG and DO obtained the funding. CG collected the data. All authors were involved in the analysis and interpretation of the data. CG drafted the article; JP, SG and DO revised it critically for important intellectual content; all authors approved the final version.
